# Prolonged Antigen Ingestion by Sensitized Mice Ameliorates Airway Inflammation

**DOI:** 10.5402/2011/818239

**Published:** 2011-12-01

**Authors:** Maria de Lourdes Meirelles Noviello, Nathália Vieira Batista, Luana Pereira Antunes Dourado, Denise Carmona Cara

**Affiliations:** Departmento de Morfologia, Instituto de Ciências Biológicas, Universidade Federal de Minas Gerais, Avenida Antônio Carlos 6627, Pampulha, 31270-901 Belo Horizonte, MG, Brazil

## Abstract

Food allergy frequently precedes or coexists with respiratory allergy, and although restriction of contacts with the allergen is the elected clinical procedure, oral immunotherapy (OIT) has proven to be surprisingly efficient in clinical trials. We investigated whether prolonged restriction and voluntary exposure of previously sensitized (immunized) mice to ovalbumin (OVA) in the drinking water would alter subsequent responses to bronchial (aerosol) challenge with OVA. We found a significant suppression of bronchial inflammation, with marked reduction of eosinophils. IL-4, CCL-2, and CCL-11 are not associated with elevation in IL-10 production or Foxp3 expression, with only minor digestive symptoms.

## 1. Introduction

Immunological activity in the gastrointestinal tract is diverse and complex. Dietary proteins and products of the microbiome constantly activate physiological interactions among immunologically relevant cells and molecules [1, 2]. The most frequent consequence of the injection of a previously ingested protein, with adjuvants, is a significant reduction in the magnitude of the resulting immune response, a phenomenon known as “oral tolerance” [2]. Oral tolerance was observed one century ago [3], but the mechanisms of this phenomenon are as yet to be clarified, in spite of intense basic and clinical research [4, 5]. Easily induced in naïve organisms, oral tolerance is difficult to be induced in primed (immunized) animals; oral exposures may actually induce secondary (booster) responses and anaphylactic reactions [4].

Food allergy frequently precedes or coexists with respiratory allergy, and respiratory exposure to particles of dietary materials may trigger asthmatic reactions [1, 6]. Previous experiments in our laboratory have shown that ingestion of the antigen by previously immunized mice may result in weight loss, elevation of specific IgE titers, and eosinophilic infiltration in the gut mucosa [7]. Nevertheless, there is evidence that prolonged restriction and voluntary ingestion of the antigen by immunized animals may also reduce immune responsiveness [8, 9].

Herein, we investigated whether these prolonged exposures would alter subsequent bronchial responses to aerosol challenge with the antigen. We found that this treatment significantly suppressed bronchial inflammation, with marked reduction of eosinophils, cytokines (IL-4), and chemokines (CCL-2 and CCL-11) in lung tissues, classically associated with respiratory allergy [10]. There was no increase in the serum titers of specific IgE, but this was not associated with elevation in IL-10 production or Foxp3 expression.

Food allergy is generally interpreted as a failure of oral tolerance [4]; however, there is also evidence that oral tolerance, rather than being an inhibition, promotes a stabilization of immune responsiveness [11, 12] by directly modifying inflammatory reactions [13]. We suggest that prolonged restriction and voluntary ingestion of the specific antigen by immunized animals may hinder allergic reactions and may be reached with minor digestive disturbances.

## 2. Material and Methods

### 2.1. Animals

8-week-old BALB/c mice, of both sexes, obtained from the animal facility at Federal University of Minas Gerais, Brazil were used. They were handled according to the rules established by the local ethical committee in animal experimentation, which are in accordance with the Ethical Principles in Animal Experimentation (Protocol CETEA 191/2007). All mice received standard (Purina) mouse chow throughout the experiment. Each experimental group contained five mice. The mice were kept during all the experimental period sharing a cage.

### 2.2. Subcutaneous Immunizations and Oral and Aerosol Exposure to OVA

Primary immunization (day 0) consisted of 0.2 mL saline containing 10 *μ*g OVA (five times crystallized hen egg albumin; Sigma, St. Louis, MO, USA) plus 1 mg Al(OH)_3_ as adjuvant; secondary immunization (day 14) consisted of 0.2 mL saline containing 10 *μ*g OVA and no adjuvant. From day 21 to 35, some mice received a 1/5 solution of filtered egg white in their drinking water (approximately 10 mg OVA/mL) as their only liquid source. Control groups drank tap water. From day 36 to 41, some mice were challenged with an aerosol of 10 mg OVA/mL; control mice received an aerosol of saline.

### 2.3. Experimental Groups

Mice in control group, although s.c immunized, was not further exposed to OVA, either orally or by aerosol; aerosol group was s.c immunized and challenged with OVA aerosol; oral group was s.c immunized and drank OVA but were not challenged by aerosol; oral/aerosol group was s.c immunized, drank OVA, and was challenged with OVA aerosol. One additional control group nonimmunized and nonchallenged was performed to verify the influence of sensitization and challenge in IgE production and normalize the expression of Foxp3 mRNA.

### 2.4. Serum Collection and Anti-OVA IgE Assay (ELISA)

At the end of the experiment, on day 42, all mice were deeply anesthetized by intraperitoneal injection (i.p.) of 10 mg/kg xylazine and 200 mg/kg ketamine hydrochloride, and blood samples were obtained for anti-OVA IgE assays. Levels of anti-OVA IgE were evaluated by capture-ELISA using plates coated with rat anti-mouse IgE, 50 *μ*L total serum, and biotinylated OVA. The results for anti-OVA IgE antibodies were reported in arbitrary units (AU) using a highly positive reference serum determined as 1000 units.

### 2.5. Bronchoalveolar Lavage (BAL)

After serum collection, the animals were euthanized by i.p. injection of more of the same anesthetic solution, and a bronchoalveolar lavage was performed. The tracheae were cannulated and lungs were lavaged with 0.5 mL saline. This procedure was repeated 3 times. Total and differential cell counts of bronchoalveolar lavage fluid were determined by hemocytometer and cytospin preparation stained with May-Grunwald/Giemsa (Merck) and cells were analyzed according to their morphology. Cell types under light microscopy were expressed as a percentage after counting 200 cells.

### 2.6. Determination of EPO Activity

The eosinophil peroxidase (EPO) assay was performed with 100 mg of tissue from each lung, homogenized in 1.9 mL of PBS, and centrifuged at 12.000 ×g for 10 min. The supernatant was discarded and the erythrocytes were lysed. The samples were then centrifuged again, the supernatant was discarded, and the pellet was suspended in 1.9 mL of 0.5% hexadecyltrimethyl ammonium bromide in PBS-saline. The samples were frozen three times in liquid nitrogen and centrifuged at 4°C at 12,000 xg for 10 min. The supernatant was used in the enzymatic assay. Briefly, o-phenylenediamine (OPD; 10 mg) was dissolved in 5.5 mL of distilled water and then 1.5 mL of OPD solution was added to 8.5 mL of Tris buffer (pH8.0), followed by the addition of 7.5 *μ*L of H_2_O_2_. Using a 96-well plate, 100 *μ*L of substrate solution was added to 50 *μ*L of each sample. After 30 min, the reaction was stopped with 50 *μ*L of 1 M H_2_SO_4_ and the absorbance was read at 492 nm.

### 2.7. Histological Analysis

Lungs were removed after BAL collection and perfused via the right ventricle with 10 mL of cold PBS to remove residual blood. The one part of left lung and the proximal jejunum were fixed in 10% formalin in PBS and processed for paraffin embedding. Histopathological sections (4 *μ*m) were stained with hematoxylin-eosin (HE). Mucosal and submucosal eosinophils of proximal jejunum were counted in 10 random fields/slide, using a 40x objective (53.333 *μ*m^2^/field) and the inflammatory exudate of perivascular and peribronchial were evaluated The right lung and the other part of the left lung was frozen immediately for EPO, ELISA, and RT-PCR measurements.

### 2.8. Cytokines and RNA Analysis

The cytokines (IL-4, IL-10) and chemokines (CCL-2, CCL-11) were measured using an ELISA kit according to the manufacturer's instructions (R&D Systems, Minneapolis, MN, USA).

Total RNA was isolated from the lungs and real-time RT-PCR was performed in an ABI PRISM 7900 sequence detection system (Applied Biosystems) using SYBR Green PCR Master Mix (Applied Biosystems) after RT of 1 *μ*g RNA using SuperScript II reverse transcriptase (Invitrogen Life Technologies). The relative level of gene expression was determined by the comparative threshold cycle method, as described by the manufacturer, whereby data for each sample were normalized to *β*-actin and expressed as a fold change compared with a group of naive mice. The following primer pairs were used for *β*-actin, GGA TGC AGA AGG AGA TTA CTG (forward), and CGA TCC ACA CAG AGT ACT TG (reverse); Foxp3, CCCAGGAAAGACAGCAACCTT (forward), and TTCTCACAACCAGGCCACTTG (reverse).

### 2.9. Statistics

All values were expressed as mean ± SEM. Parametric data were evaluated using analysis of variance, followed by the Tukey test for multiple comparisons. Differences were considered statistically significant at *P* < 0.05. The GraphPad Prism software was used.

## 3. Results

### 3.1. Alterations in the Digestive Tract

**Figure 1:**
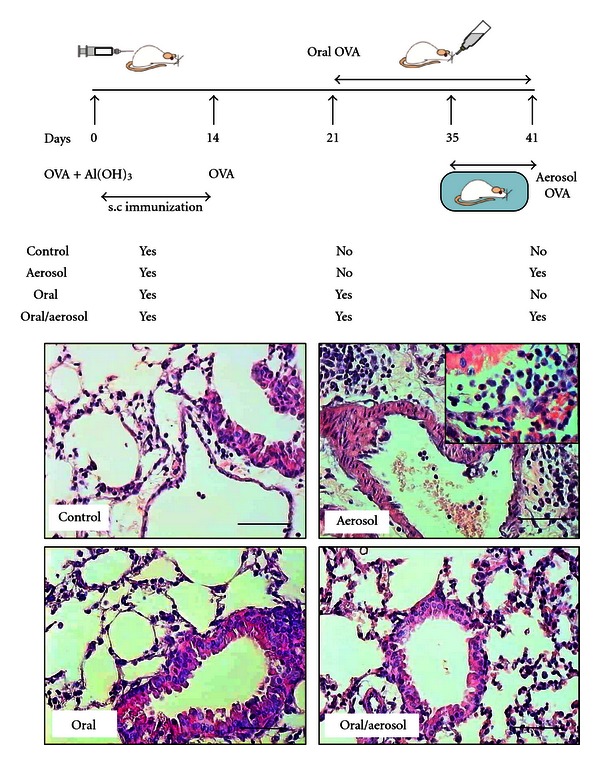
Schematic protocol for oral and aerosol challenge in sensitized mice and lung histology. BALB/c mice were sensitized with ovalbumin (OVA) in Al(OH)_3_ (day 0) and with OVA (day 14). They were subjected to drink water (control group and aerosol group) or OVA (oral group and oral/aerosol group) for 20 days (days 21 to 41). For 6 days (days 36 to 41), mice (aerosol group and oral/aerosol group) were aerosolized with OVA or saline (control group and oral group). Lung HE staining for each group is shown. Scale bar means 20 *μ*m.

Four experimental groups were used, as shown in Figure 1. Prolonged ingestion of OVA by previously s.c. immunized mice induced a mild intestinal inflammatory response with increased eosinophil infiltration in the proximal jejunum; as measured by eosinophils/field in the different groups: control = 2.1 ± 0.3, aerosol = 1.9 ± 0.4, oral = 6.2 ± 0.8, oral/aerosol = 5.5 ± 0.7. No mucosal edema, loss of mucosal architecture or epithelial erosion occurred in any of the groups (data not shown). The egg white consumption by each mouse was estimated as 3.3 mL/day (33 mg OVA). 

### 3.2. Prolonged Ingestion of Ovalbumin Prevented the Influx of Inflammatory Cells into the Lungs and the Increase of Serum Anti-OVA IgE after Reexposure to OVA by Aerosol

**Figure 2:**
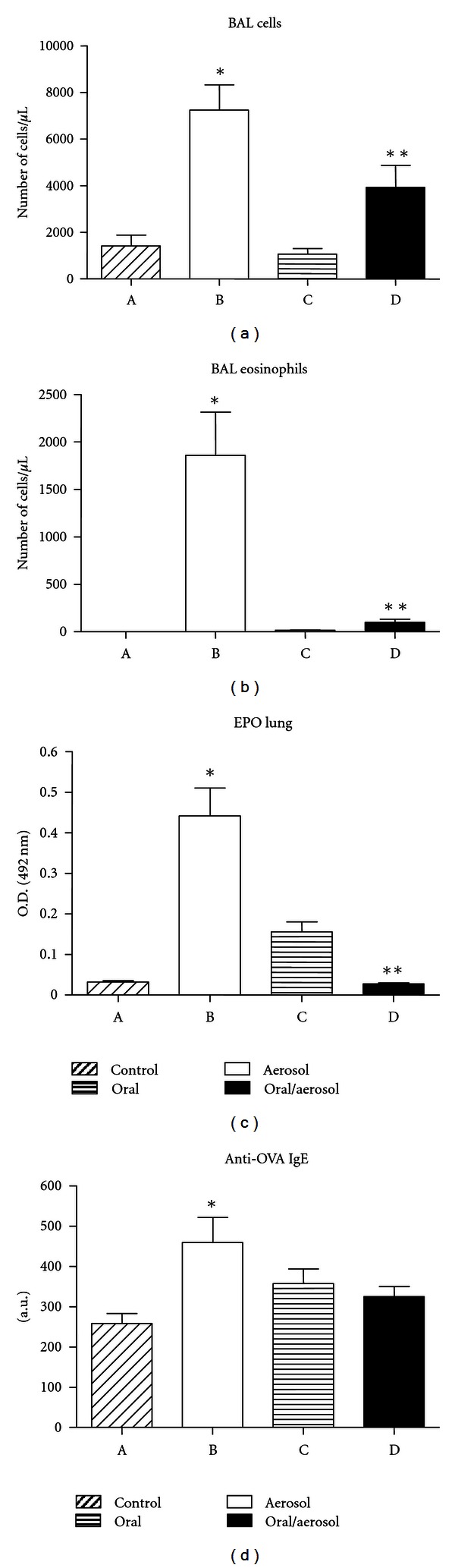
Leukocyte recruitment to the bronchoalveolar space and determination of serum ovalbumin-specific IgE. BALB/c mice were sensitized with ovalbumin (OVA) in Al (OH)_3_ (day 0) and with OVA on day 14. They were subjected to drink water (control group and aerosol group) or OVA (oral group and oral/aerosol group) for 20 days (days 21 to 41). For 6 days (days 36 to 41), mice (aerosol group and oral/aerosol group) were aerosolized with OVA or saline (control group and oral group). (a) Total cells present in the BAL (b) airway eosinophilia, (c) activity of EPO in lungs, and (d) serum ovalbumin-specific IgE. Values represent the means +/− SEM, *n* = 5, representative of 2 experiments; *significant difference (*P* < 0.05) aerosol group versus control group, ** aerosol group versus oral/aerosol group.

Prolonged exposure to ovalbumin (OVA) in the drinking water of previously sensitized (immunized) mice, altered subsequent responses to bronchial (aerosol) challenge with OVA. Mice were concomitantly subcutaneously (s.c.) immunized with OVA, exposed to OVA in the drinking water and subsequently exposed to OVA in aerosol (Figure 1, oral/aerosol group). Control animals were s.c immunized but not exposed to OVA subsequently (Figure 1, control group) or only exposed to OVA in aerosol or orally (Figure 1, aerosol and oral groups). As expected, evaluation of lung inflammation showed that mice not exposed to OVA aerosol had normal lung histology (Figure 1); mice challenged with OVA aerosol in the absence of oral OVA exposure, had an accumulation of inflammatory cells in perivascular areas (Figure 1) consisting mainly of eosinophils (Figure 1, higher magnification). However, mice exposure to OVA by the oral route and then being challenged with OVA aerosol showed a significant reduction in lung inflammation (Figure 2(d)). This was also observed in the total numbers of recruited cells (Figure 2(a)) and eosinophils (Figure 2(b)) into the alveolar space. Moreover, EPO activity confirmed reduced lung eosinophils levels in group oral/aerosol (Figure 2(c)). 

Also as expected, higher anti-OVA IgE levels were found in OVA immunized mice (Figure 2(d), control group) compared with naive mice, which had titers 48.76 ± 9.04 a.u. Further increased titers were found in mice challenged with OVA aerosol in the absence of oral OVA exposure (Figure 2(d), aerosol group), but not in mice not exposed to OVA aerosol (Figure 2(d), control and oral groups). Thus, the oral exposure to OVA significantly aborted the increase in anti-OVA IgE triggered by OVA aerosol (Figure 2(d), oral/aerosol group). 

### 3.3. Effects on the Expression of IL-4, IL-10, Chemokines CCL-11, CCL-2, and Foxp3 mRNA

**Figure 3:**
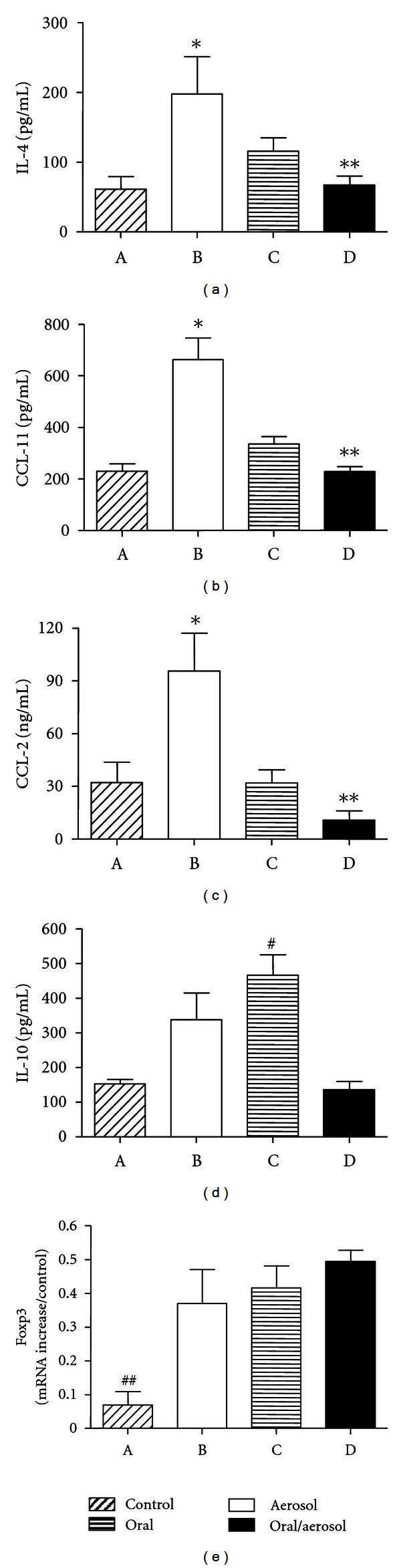
Lung IL-4, CCL-2, CCL-11, IL-10 levels, and Foxp3 mRNA expression. BALB/c mice were sensitized with ovalbumin (OVA) in Al(OH)_3_ (day 0) and with OVA on day 14. They were subjected to drink water (control group and aerosol group) or OVA (oral group and oral/aerosol group) for 20 days (days 21 to 41). For 6 days (days 36 to 41), mice (aerosol group and oral/aerosol group) were aerosolized with OVA or saline (control group and oral group). (a) Levels of IL-4. (b) Levels of CCL-2. (c) Levels of CCL-11. (d) Levels of IL-10. (e) Increased expression of Foxp3 mRNA. Values represent the means +/− SEM, *n* = 5, representative of 2 experiments, *significant difference (*P* < 0.05) aerosol group versus control group, ** aerosol group versus oral/aerosol group, # oral/aerosol group versus oral group, and ## control group versus aerosol, oral and oral/aerosol groups.

Aerosol antigen challenge induced the expression of IL-4 (Figure 3(a)), CCL-2 (Figure 3(b)), and CCL-11 (Figure 3(c)) in the lung of sensitized mice, which are important mediators in the infiltration of inflammatory cells during allergen-induced pulmonary inflammation [10]. This increase was not observed in the group orally exposed to OVA concomitantly with the aerosol challenge (Figure 3). We further examined if reduction of inflammation could be associated with increased production of IL-10 or increase in regulatory cell expressing Foxp3, but no such correlation was found; indeed significant increase in IL-10 was found only in the group subjected to prolonged ingestion of OVA in the absence of OVA aerosol (Figure 3(d)). Foxp3 mRNA expression increased after challenge by OVA aerosol or oral OVA, but no significant differences were found when comparing all groups challenged with OVA (Figure 3(e)).

## 4. Discussion

Our results show that the consumption of an antigen to previously immunized mice causes mild intestinal signs but is able to decrease respiratory signs induced by the same antigen. Herein we discuss that this response is probably related to a novel alternative procedure utilized in clinical trials for food allergy, named oral immunotherapy [14, 15]. Also, our results are according with other experimental data using mucosal challenge after immunization [16].

Food allergy is a difficult clinical problem for which there is no available efficient therapy. Rigorously controlled clinical trials are currently underway, but important unanswered questions remain [17]. This applies both to food allergy [1] and to relations with the gut microbiome, in which the problem is to distinguish between commensal and pathogenic microbes [2].

Studies of oral tolerance to purified proteins in mice have shown that ingestion of a T-dependent potent immunogen, followed by s.c. immunization with the same protein in adjuvant, reduces specific antibody production in rates that are inversely proportional to the ingested dose of antigen. When high doses are ingested, antibody responses are significantly inhibited, but when lower doses are ingested, a residual formation of antibodies persists. Strikingly, in these partially tolerant mice even repeated booster stimulation with the antigen in adjuvant fail to alter the established levels of antibody response. Thus, rather than simply a route to inhibition the oral route favors a robust stabilization of specific antibody formation [11].

These changes are easy to establish in naïve mice, but become progressively more difficult to attain with time after parenteral immunization [8]; however, there is evidence, such as that shown herein, that prolonged ingestion of the antigen may hinder the progressive quality of secondary antibody responses, even after further immunization. A particular aspect of these changes, that is relevant for clinical studies, is the pronounced effect upon regional eosinophil infiltration and the formation of specific IgE.

 In our experiments, animals in the aerosol group that did not ingest the antigen displayed intense bronchial eosinophilic infiltration and high eosinophilic peroxidases (EPO) and specific IgE activity; elevated IL-4, CCL-11, and CCL-2 are associated with airway inflammation [10]. On the other hand, animals that ingested the antigen, although previously immunized and forming specific IgE, displayed milder bronchial inflammation.

At present, no explanation is available for these results. Both in humans and animal models, including allergic conditions, parasite infections and inherited immunodeficiencies eosinophilia and enhanced IgE production are linked to helper T cell oligoclonal expansions, suggesting a possible link between immunopathology and a reduction in clonal diversity [18]. There is also no accepted explanation for oral tolerance, but it is conceivable that immunological activity in the gut, which is constantly dealing with dietary materials and the commensal microbiome, involves a wide diversity of lymphocyte clones and favors the connectivity among lymphocyte clones and thus hinders progressive and potentially pathogenic expansions of isolated lymphocyte clones [12].

The pattern of antigen ingestion by previously immunized animals also seems to affect their consequences. As previously shown by others [19], allergic responses in the gut increase bronchial inflammation when mice are challenged by gavage on alternate days. In our protocol, the animals ingested the antigen continuously as their sole liquid intake [7]. Continuous ingestion of antigen is more efficient for oral tolerance induction than a single or multiple gavages [20]. Our laboratory has studied effects of prolonged oral administration of the specific antigen to immunized mice in several situations. These animals display a strong aversion to ingest solutions containing the antigen—for example, (OVA) [21–23]. However, if submitted to ingest them as their only source of liquid for long periods, they display a mild intestinal inflammatory response with increased eosinophil infiltration in the proximal jejunum with no further serious evident damage [7]. If there was no aversion phenomenon, the gut damage could be more severe. Furthermore, the prolonged uninterrupted intake of antigen not only reduced bronchial inflammation, but also reduced other symptoms of aggression, such as weight loss and erosion of the gut epithelium associated with allergic reactions in the gut (data not shown). Actually, the relative rarity of gut allergy in daily living is surprising due to the large variety of potential allergens contacting the gut. It is conceivable that this protection derives exactly from the connectivity among the wide variety of activated lymphocytes and immunoglobulin production in the gut mucosa, which hinders oligoclonal expansions. Clinical protocols of oral immunotherapy (OIT) of allergic patients use daily and increasing doses of the allergen, and this favors the establishment of tolerance to eventual contacts independently of the periods of abstinence; it remains to be established whether this tolerance is transient or permanent. [17]. In our model, the suppressive effect persisted even after oral challenge with ovalbumin was stopped seven days before the animals were submitted to aerosol challenge (data not shown).

Among the many mechanisms proposed to explain oral tolerance, the activation of regulatory cells expressing Foxp3 and the secretion of suppressive cytokines, such as IL-10, have been extensively studied [4, 24]. There are data suggesting that Foxp3 expressing cells proliferate in inflammatory conditions [25] and IL-10 have a role in the perpetuation of allergic inflammation [26]. In our experiments, however, the suppression of inflammation was not related to these factors (see Figures 3(d) and 3(e)). 

Our results point to two conceptual watersheds. One is empirical and shows that prolonged intake of the antigen favors tolerance and reinforces equally empirical trials with OIT. The other is theoretical and suggests that we might switch from theories preoccupied with the intensity of immune responses to those concerned with the diversity of clonal activation as an important variable in immunopathology [12, 18].
